# Women and Their Victims

**Published:** 1888-12-15

**Authors:** 


### Editor's Letter-Box.

[Our Correspondents are reminded that prolixity is a great bar to publication, and that brevity of style and conciseness of statement greatly facilitate early insertion.]

### WOMEN AND THEIR VICTIMS.

I feel sure that if women could witness the misery and pain caused by the ruthless destruction of birds, they would one and all refrain from any action that could encourage such wanton cruelty. A well-known writer remarks that "lack of imagination is the great defect of the characters of the poor;" but others may lack imagination also, and I believe that this accounts, in no small measure, for the apathy displayed by women on a subject in which they might be supposed to be interested. I beg you will allow me to thank you for bringing forward this matter and for speaking of it in no uncertain voice. I belong to that large class of busy wives and mothers whose sympathies and intellects are keenly alive to such questions. We would almost as readily join a society for the perpetration of cruelty towards children as wear decorations which tell of cruelty to the young of birds; but we are not credited with any good motive in our abstention from such things, but probably merely set down as "dowdy" and "unstylish." None the less, sir, we pray for "all that touches life with upward impulse," and we rejoice over all that broadens the minds and enlarges the sympathies of our fellow-women. Perhaps the careers of devoted work now open to some of the noblest women tend to make an apparent line of demarcation between them and those of us who are living an ordinary life, and it is not the least hard part of our daily task that we have not time to express our concurrence with the manly words so often to be found in "The Doctor's Pulpit" and other portions of your valuable paper.

A Houseworker.

